# Defining the vaccination window for respiratory syncytial virus (RSV) using age-seroprevalence data for children in Kilifi, Kenya

**DOI:** 10.1371/journal.pone.0177803

**Published:** 2017-05-22

**Authors:** Joyce U. Nyiro, Ivy K. Kombe, Charles J. Sande, James Kipkoech, Patience K. Kiyuka, Clayton O. Onyango, Patrick K. Munywoki, Timothy M. Kinyanjui, D. James Nokes

**Affiliations:** 1 KEMRI-Wellcome Trust Research Programme, Centre for Geographic Medicine Research-Coast, Kilifi, Kenya; 2 Department of Paediatrics, University of Oxford, Oxford, United Kingdom; 3 Kenya Medical Research Institute/ Centre for Disease Control and Prevention, Kisumu, Kenya; 4 School of Mathematics, University of Manchester, Manchester, United Kingdom; 5 School of Life Sciences and WIDER, University of Warwick, Coventry, United Kingdom; Imperial College London, UNITED KINGDOM

## Abstract

**Background:**

Respiratory syncytial virus (RSV) is an important cause of lower respiratory tract disease in early life and a target for vaccine prevention. Data on the age-prevalence of RSV specific antibodies will inform on optimizing vaccine delivery.

**Methods:**

Archived plasma samples were randomly selected within age strata from 960 children less than 145 months of age admitted to Kilifi County Hospital pediatric wards between 2007 and 2010. Samples were tested for antibodies to RSV using crude virus IgG ELISA. Seroprevalence (and 95% confidence intervals) was estimated as the proportion of children with specific antibodies above a defined cut-off level. Nested catalytic models were used to explore different assumptions on antibody dynamics and estimate the rates of decay of RSV specific maternal antibody and acquisition of infection with age, and the average age of infection.

**Results:**

RSV specific antibody prevalence was 100% at age 0-<1month, declining rapidly over the first 6 months of life, followed by an increase in the second half of the first year of life and beyond. Seroprevalence was lowest throughout the age range 5–11 months; all children were seropositive beyond 3 years of age. The best fit model to the data yielded estimates for the rate of infection of 0.78/person/year (95% CI 0.65–0.97) and 1.69/person/year (95% CI 1.27–2.04) for ages 0-<1 year and 1-<12 years, respectively. The rate of loss of maternal antibodies was estimated as 2.54/year (95% CI 2.30–2.90), i.e. mean duration 4.7 months. The mean age at primary infection was estimated at 15 months (95% CI 13–18).

**Conclusions:**

The rate of decay of maternal antibody prevalence and subsequent age-acquisition of infection are rapid, and the average age at primary infection early. The vaccination window is narrow, and suggests optimal targeting of vaccine to infants 5 months and above to achieve high seroconversion.

## Introduction

Acute respiratory infection (ARI) is a leading cause of morbidity and mortality in children <5 years old worldwide [1], and respiratory syncytial virus (RSV) is the most important viral pathogen responsible for annual bronchiolitis and pneumonia epidemics in these young children [2–5]. Global estimates indicate that RSV may cause about 0.3 million deaths in young children per year and 99% of these occur in low-income countries[1].

To date there are no licensed vaccines for prevention of RSV disease in infants and young children. Some candidate vaccines have shown promising results[6–8]. Most recently, an attenuated vaccine MEDI ΔM2-2, developed by the use of reverse genetics systems has been shown to be highly restricted in replication and more immunogenic in RSV seronegative children than the previous lead live attenuated RSV vaccine candidates [9]. As a result, these findings provide evidence of availability of a promising candidate vaccine for young children and infants in the near future.

The primary target for RSV vaccination is children under 6 months of age; a group highly susceptible to severe RSV disease[10]. However, vaccination of this age group is complicated by the presence of maternal antibodies, among other factors[11]. Assuming the efficacy of a potential vaccine is significantly reduced if administered in the presence of maternal antibodies, it follows that the age of vaccination should be delayed. However, a majority of primary RSV infections are acquired early in life[12] and so delaying vaccination could result in missing out on a large proportion of preventable infections. As such a vaccination window or age should be established such that there is minimal maternal antibody interference and a majority of infections have not occurred.

Analysis of an age specific seroprevalence profile could inform on the age distribution at attack of the disease in a given population. Data from previous serological studies of RSV specific antibodies suggest early age-acquisition of RSV specific antibodies following decay in maternal antibody[13, 14]. RSV seroprevalence has been found to increase rapidly with age reaching over 90% by three years of age and attaining 100% seropositivity by 5 years with mean duration of maternal antibody estimated to be 3.3 months[14]. However, RSV antibody dynamics could potentially complicate the interpretation of an age specific seroprevalence profile. The presence of maternal antibodies up to about six months of age might interfere with antibody acquisition through infection in the first few months of life[15, 16]. In addition, studies have shown that both neutralizing and total RSV specific antibodies acquired during primary infection wane to pre-infection levels over time, about one year for total antibody and three months for functional antibodies[15, 17]. A direct analysis of the seroprevalence profile without an attempt at modelling these antibody dynamics could result in an underestimation of the true rate of infection and the age of primary infection.

Catalytic models explain antibody dynamics as a function of age and use age-stratified serological data to estimate the force of infection. The simple catalytic model can be modified to allow for varied structures for non-immunizing infections[18], and different forms of the force of infection function[19–21]. Previously, catalytic models have been used to provide estimates of the rate of decay of maternal antibodies and the per capita rate at which susceptible individuals acquire infection (the force of infection) [19, 20, 22, 23]. Subsequently, one can establish the average age at primary infection and a “window” of vaccination.

In this study we present the age-specific prevalence of antibodies to RSV from a rural community at the Kenyan coast. We then develop three nested catalytic models that explore different assumptions on the RSV specific antibody dynamics. Samples were selected randomly from pediatric admissions to a County hospital in coastal Kenya, and screened for antibodies to RSV by ELISA. The data from this study provides basic understanding on natural response to RSV infection and has a bearing on the optimal age of RSV vaccine delivery.

## Materials and methods

### Study site and population

We used data and archived plasma samples from children aged 1 day to less than 12 years (i.e. <145 months) who were admitted to Kilifi County Hospital (KCH) paediatric wards between 2007 and 2010 (inclusive). The County Hospital, previously known as Kilifi District Hospital (KDH), is located in a rural area along the Kenyan Coast. The Kenya Medical Research Institute (KEMRI)-Wellcome Trust Research Programme provides clinical care on the hospital paediatric wards and runs the Kilifi Health and Demographic Surveillance System (KHDSS) covering an area of approximately 900km^2^ which is within 50km north, 50km south, and 30km west of the hospital, and includes a population (2010 enumeration) of approximately 260,000[24, 25]. Patients admitted to the hospital undergo a standard medical review, have an admission blood collected and are identified on the KHDSS population register [24].

The study participants were selected at random from Kilifi Integrated Data Management System (KIDMS) admission register regardless of diagnosis and stratified by age into one month age groups up to 11 months, then 12-<15m, 15-<18m, 18-<24m, 24-<36m, 36-<60m, 60-84m, 84-<108m and 108<145m. These children were linked to stored serum samples collected on admission, which were retrieved and an aliquot screened for the presence of antibodies to RSV. Being a resident of KHDSS, admitted to KCH between 2007 and 2010 and having a stored plasma sample at admission were inclusion criteria. All re-admissions were excluded, i.e. the total number of samples in the present analysis is exactly the total number of study participants.

To reduce bias due to seasonal transmission and in order to provide an average seroprevalence in the presence of seasonal and longer-term variation in transmission, cross-sectional sampling covered a period of four years. Each year was divided into quarters with each quarter contributing 60 samples such that each of the 20 age groups had 3 samples. This gave a total of 960 samples for the 4-year period, 240 samples from each year. Of the 960 samples each of the 20 age groups had 48 samples, 12 collected each year. Sample size was estimated by standard techniques to provide seroprevalence precision estimates of 50% within a width of +/-14%.

### Ethical approval

All parents and guardians gave written consent to have their children participate in the paediatric inpatient surveillance at KCH and for storage of blood samples for use in future research. The KEMRI Ethical Review Committee approved this study.

### Laboratory procedures

All admission plasma samples were tested for antibody concentration with an IgG based ELISA method using crude virus extract from lab adapted RSV A2 culture and specific antibody concentrations recorded as log arbitrary units as determined by a local standards procedure. The optimal dilutions for RSV-A2 antigen and serum were determined by a checkerboard titration. The crude virus RSV lysate preparation was as previously described by Ochola et al [16]. The RSV IgG ELISA procedure was performed as described previously [16].

The samples were run in duplicate to account for any variability in the assay operation such as caused by pipetting errors. Within every plate, both high and low controls were run alongside the samples. A graph was plotted over time to check for both the standards and coating antigen deterioration.

A pooled standard serum from adults, serially diluted in 2 fold dilutions from 1:50 to 1:2800 was run in each plate to generate a standard curve. The OD values of the standard sera dilutions were assigned arbitrary unit (AU) values. Standardized arbitrary units (and log_10_ transformed) of RSV specific IgG for samples were estimated by interpolation of a standard curve generated from the pooled adult serum (serum standard) tested in each ELISA run.

**Fig 1:**
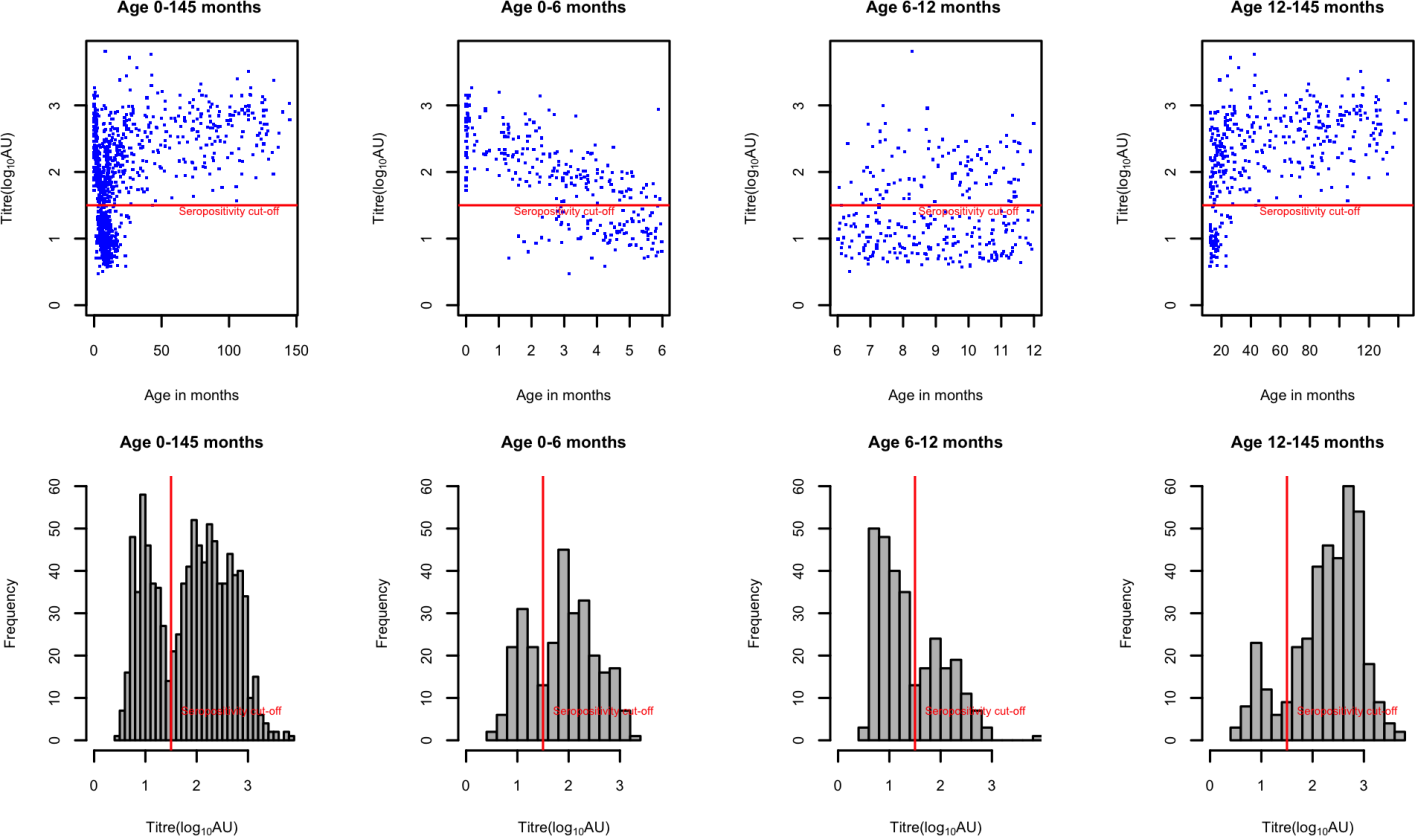
Plots of RSV antibody titers. Top row: Scatter plots of antibody titer level by age groups. Bottom row: Histograms of antibody titers by age groups. Red lines show the 1.5 cut-off used to define seropositivity.

A frequency distribution of log_10_AU values for all sera screened was made to determine a suitable cut off between seropositive and seronegative status. A cut off point of 1.5 as previously applied by Ochola et al, [16] was used in this study to allocate RSV serostatus as this cut off was also found to delineate the positives and negatives in the bimodal distribution in this study, as shown in (Fig 1).

### Statistical analysis

Data were analyzed using STATA version 11 (StataCorp, College Station, Texas, USA). The proportions of samples in each age class were derived with 95% confidence intervals.

### Modelling the risk of primary RSV infection

We estimated the titer-related risk of primary RSV infection and rate of loss of maternal antibodies concurrently, using a nested catalytic model built up from a simple model. The nested model allows for exploration of different assumptions on population level antibody dynamics. We assumed that antibodies were acquired through maternal transfer or exposure to infection.

A catalytic model is a population level model; individuals are grouped into different states (compartments) depending on assumptions in the model. The simple catalytic model had two compartments: (i) the proportion susceptible and seronegative at each age group, *a*, i.e. *S(a)*, and (ii) the proportion (ever) infected, by age, *F(a)*. The ‘(ever) infected’ individuals encompass the infected and recovered individuals. The parameter estimated is the per capita rate of infection per year (force of infection), e.g. if the estimate is given as 0.5/person/year, this means that everyone experiences an infection pressure equivalent to 0.5 infections in a year (1 infection every 2 years). To account for presence of maternal antibodies, an *M(a)* compartment and a parameter for the rate of decay of maternal antibodies were introduced. In this case the model assumed that presence of maternal antibody is refractive to infection. The *M(a)* compartment was then split into 2, changing the distribution of the duration of maternally acquired antibodies from Exponential to Erlang with a shape parameter equal to 2. This modification means that longer durations of maternal antibodies have higher probabilities than shorter durations.

**Fig 2:**
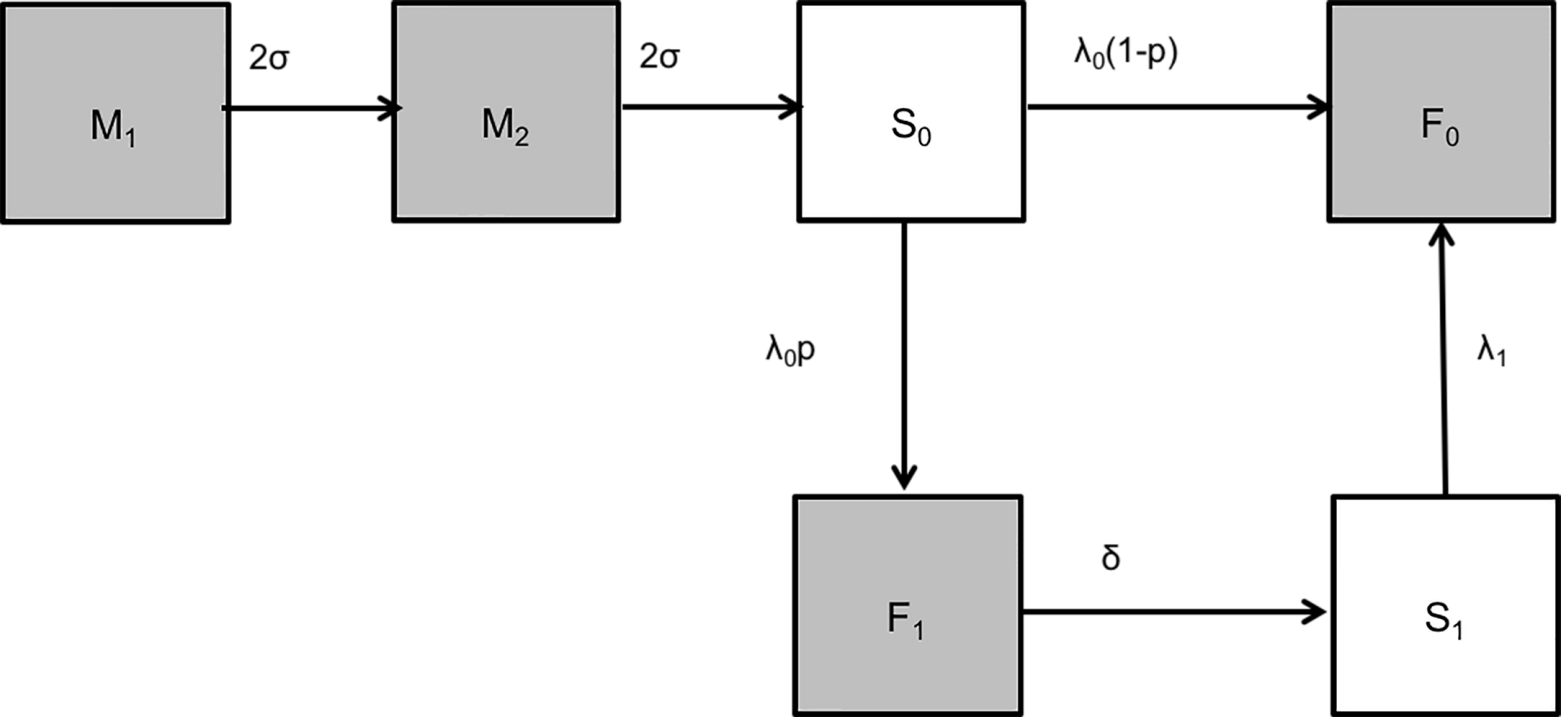
The nested model structure for exploring age-prevalence data for RSV using catalytic infection models. The compartments in the model represent the following states of the population: M = Individuals with maternally acquired antibodies (split into 2, M_1_ and M_2_ to allow for improved fit), S_0_ = Seronegative after loss of maternally acquired antibodies, F_0_ = Permanent seropositive status after infection, F_1_ = Temporary seropositive status after primary infection, S_1_ = Seronegative after loss of infection acquired antibodies. When p = 0, the model reduces to MSF, when p = 1 and only 1 rate of infection is estimated *(λ*_*0*_ *= λ*_*1*_) the model is MSFSF_1_ and with p = 1 and 2 rates of infection estimated *(λ*_*0*_ *≠ λ*_*1*_) the model is MSFSF_2_.

We explored three main forms of the nested model structure (Fig 2) that explore the possible effects of transient RSV antibody following primary infection and, implicitly, age-related per person incidence (i.e. age-related force of infection), as described:

#### MSF

At age zero, all individuals are seropositive for maternally acquired RSV antibodies, the M compartment. They lose their maternally acquired seropositive status at a constant rate σ entering the susceptible compartment S_0_ in which they become infected at a constant rate λ_0_ and enter the F_0_ class of (ever) infected. In F_0_ they experience subsequent infections, at an unknown rate, while seropositive.

#### MSFSF_1_(λ_0_ = λ_1_)

This is an extension of the MSF model where a proportion, p = 1, will get infected at a rate λ_0_ and enter the F_1_ compartment, but subsequently lose their seropositive status at a rate δ and join the S_1_ class of susceptibles. From S_1_ they get re-infected at a rate λ_1_ and move into the F_0_ class following reinfection and experience subsequent infections while seropositive.

#### MSFSF_2_(λ_0_ ≠ λ_1_)

An extension of the *MSFSF*_*1*_*(λ*_*0*_ *= λ*_*1*_*)* but with the primary force of infection λ_0_ not equal to the secondary force of infection λ_1_.

The model structure is shown in (Fig 2) and the parameters as described in Table 1. The nested structure presented assumes that the presence of maternal antibodies is refractive to infection, which might not necessarily be the case. We later modify this assumption by allowing infection in the M compartments. Also a different cut-off for seropositive status for ages 6 months and below was explored. We allowed for an age-dependent force of infection function. The function chosen was stepwise for ages 0–1 year and 1–12 years in the MSF and MSFSF_1_ models. Justification for the steps can be found in S1 Text. Age dependence is implicit in the MSFSF_2_ model. In addition, birth and death processes were ignored.

**Table 1:** Description of the model parameters.

Parameter	Description	Value
**p**	Proportion that loses antibodies after primary infection.	0 or 1
**δ**	Rate of loss of antibodies post primary infection	4/person/year [15, 17]
**σ**	Rate of loss of maternal antibodies	Fitted
**λ**_**0**_	Rate of primary infection (primary force of infection)	Fitted
**λ**_**1**_	Rate of secondary infection (secondary force of infection)	Fitted

Parameter fitting was done using maximum likelihood estimation (MLE)[24] in Matlab version 7 (MathWorks, Massachusetts, USA). The ODEs for the model were solved numerically using the *ode45* function, which is based on an explicit Runge-Kutta (4,5) formula, the Dormand-Prince pair. Optimization was performed using the *fmincon* function, which applies constrained nonlinear optimization. Two of the models in the nested structure have the same number of estimated parameters; as such model comparison was done using the second order Akaike information criterion (AIC_C_). Confidence intervals on the parameters were established by re-fitting the model to a set of bootstrapped samples of the data. Resampling was done 1000 times and the 95% credible interval established using the 2.5^th^ and 97.5^th^ percentiles[25].

Using the parameters estimated, we calculated the average age at primary infection *A*, using the formula $A=\frac{1}{\sigma }+\frac{1}{\hat{\lambda }}$ where $\hat{\lambda }=\frac{\left(\lambda_{0-1}\times 12)+(\lambda_{1-12}\times 8\right)}{20}$, for 12 age groups between 0 and 1 year and 8 age groups between 1 and 12 years, and the optimal age to vaccinate, Av, using the formula $Av=\frac{\ln\sigma -\ln\hat{\lambda }}{\sigma -\hat{\lambda }}$ a modification of the original[26]. Here we define the optimal age to vaccinate as the age where there will be minimal maternal antibody interference and a majority of infections will not have occurred, as determined by the seropositive status.

## Results

### Description of study participants

There were 960 children selected from the admission register stratified into 20 age groups with each group having 48 children. Four hundred and one (41.8%) were female. Based on primary diagnosis at discharge, 298 (31.0%) were admitted with lower respiratory tract infection (LRTI), 181 (18.8%) with gastroenteritis, 174 (18.1%) with other conditions (cardiac problem, Burkitt’s lymphoma, epilepsy, hydrocephalus), 145 (15.1%) other infectious diseases, 57 (5.9%) malaria, 39 (4.1%) malnutrition, 37 (3.9%) congenital diseases, 17 (1.7%) anemia, while 12 (1.2%) had a missing diagnosis. Of those with a diagnosis of LRTI, 16% (5% of study participants) had an antigen confirmed RSV infection [27, 28].

### Age specific seroprevalence of RSV antibodies

Overall seroprevalence was 65.7% (95% CI: 62.7–68.7). There was 100% seroprevalence in all children less than 1month old. Seroprevalence declined from 91.7% at 1 month to a low of 25% at 6 months. Thereafter, there was slow rise between 6 months to 9 months and then a rapid change in seroprevalence from 41.7% (11 to <12 months), 52.1% (12 to <15 months), 56.3% (15 to <18 months), 83.3% (18 to <24 months), 93.8% (24 to <36 months) and 100% (36 to <60months). All children above 3 years were seropositive. The proportion seropositive per age group is shown in Table 2.

**Table 2:** Proportion of children seropositive for RSV antibodies per age group.

Age group in months	RSV IgG ELISA(%)	95% CI
0-<1	100	1
1-<2	91.67	83.56–99.78
2-<3	75	62.29–87.71
3-<4	64.58	59.88–85.96
4-<5	45.83	31.21–60.45
5-<6	27.08	14.04–40.12
6-<7	25.00	12.29–37.71
7-<8	39.58	25.23–53.93
8-<9	35.42	21.38–49.45
9-<10	43.75	29.19–58.31
10-<11	39.58	25.23–53.93
11-<12	41.47	27.2–56.13
12-<15	52.08	37.42–66.74
15-<18	56.25	41.69–70.81
18-<24	83.33	72.40–94.27
24-<36	93.75	86.65–1.01
36-<60	100	1
60-<84	100	1
84-<108	100	1
108-<145	100	1

Each age group had a total of 48 children.

### Estimates for incidence of primary RSV infection, average age of infection and optimal age for vaccination

**Fig 3:**
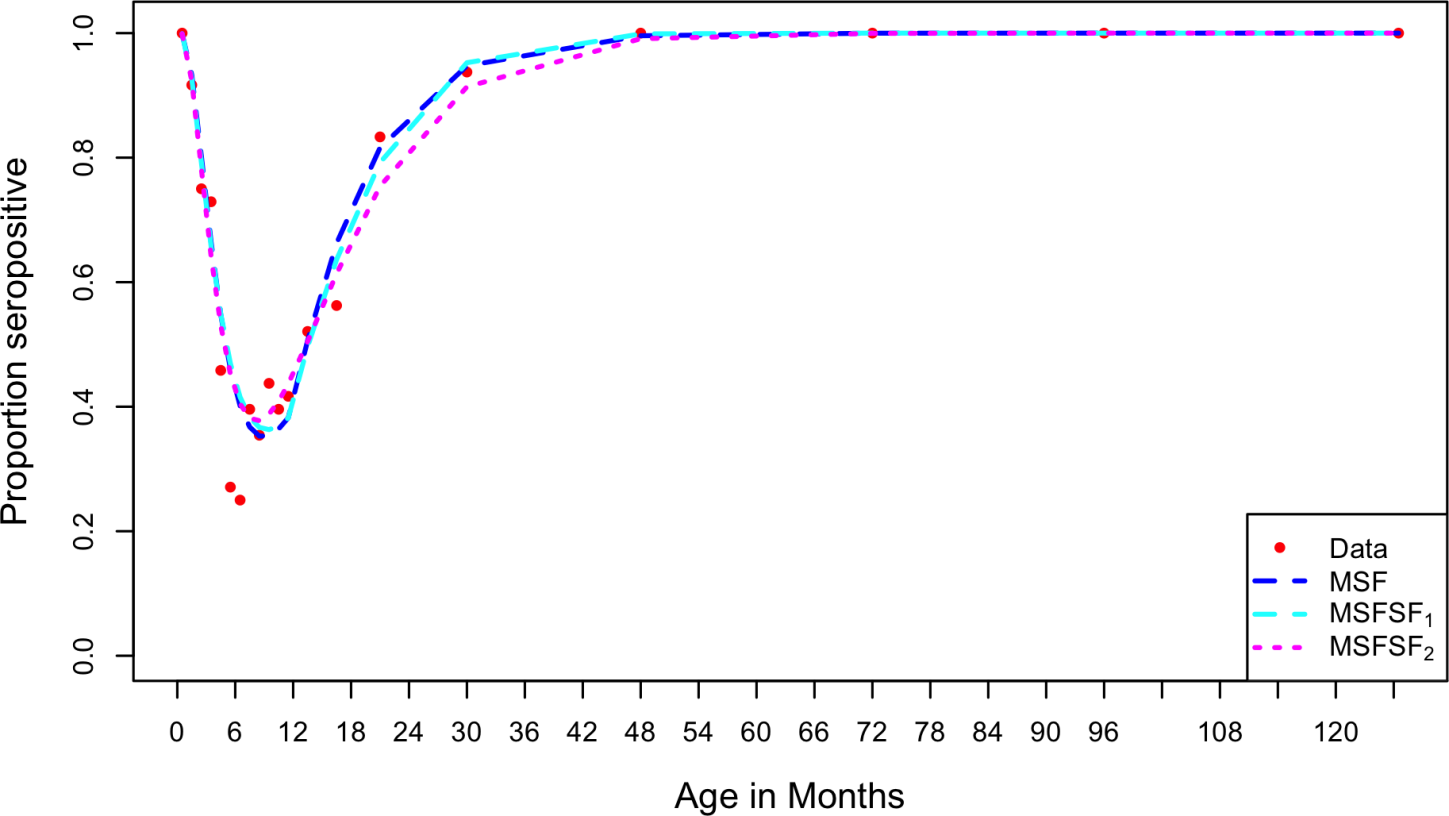
Plot of the nested models output compared to the data. The MSF model fit the data best with an AIC_C_ value of 634.6, the MSFSF_1_ had an AIC_C_ of 635.6 while the MSFSF_2_ had 637.9

We explored different assumptions in three nested catalytic models and compared the fits to data; the comprehensive results are shown in S1 Table in S1 Text. Details of the model selection process are described in S1 Text. In brief, the data supported the use of two M classes rather than one and a stepwise force of infection function rather than a constant. Of the three nested models, the simplest model (MSF) fit the data best with the least negative log likelihood and AIC_C_ values. Results are shown in Table 3 and (Fig 3).

**Table 3:** Results of fitting the three models contained in the nested model structure.

	Parameters					-LL	AIC_C_
Model	p*	δ*	σ	λ_0_	λ_1_		
**MSF**	0	NA	2.54	0.78, 1.69	NA	314.3	634.6
**MSFSF**_**1**_	1	4	2.71	1.80, 3.10	1.80, 3.10	314.8	635.6
**MSFSF**_**2**_	1	4	2.78	1.53	3.98	316.0	637.9

p = proportion that loses antibodies post primary infection; δ = rate of loss of antibodies post primary infection; σ = rate of loss of maternal antibodies; λ_0_ = primary force of infection; λ_1_ = secondary force of infection; -LL = the negative log likelihood value, the lower the value the better the model; AICc = the second order Akaike information criterion.

* These parameters are fixed (not estimated). All rates are per person per year.

**Fig 4:**
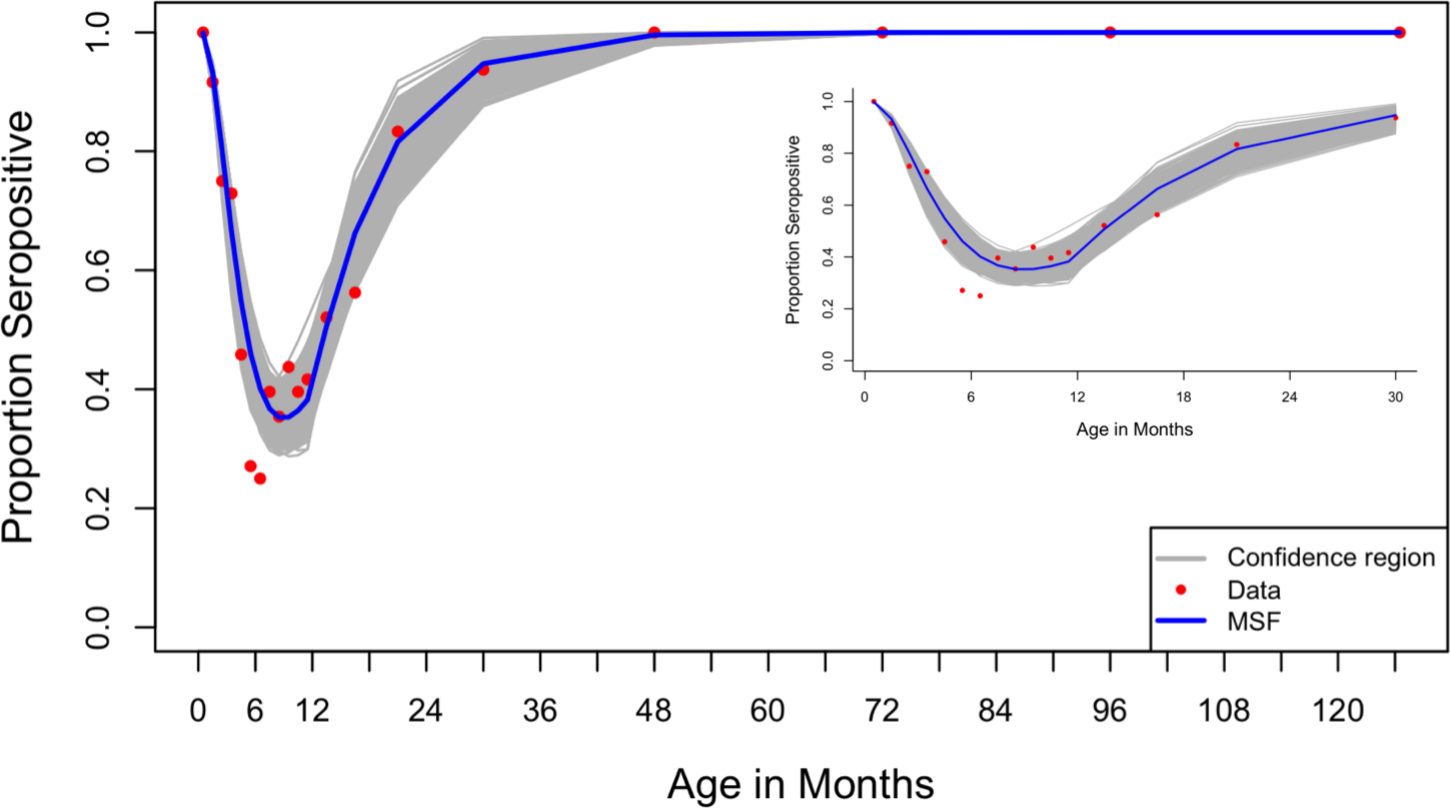
Results of the MSF model fit and the confidence region. Main: Of the three models in the nested model structure, the MSF model gave the best fit to the data and is shown by the blue line. The parameters were re-estimated to obtain the fits that gave the 95% confidence region by Bootstrapping method, grey lines. The red circles show the proportions seropositive by age group according to the data. Inset: A magnification for age range 0–3 years.

**Fig 5:**
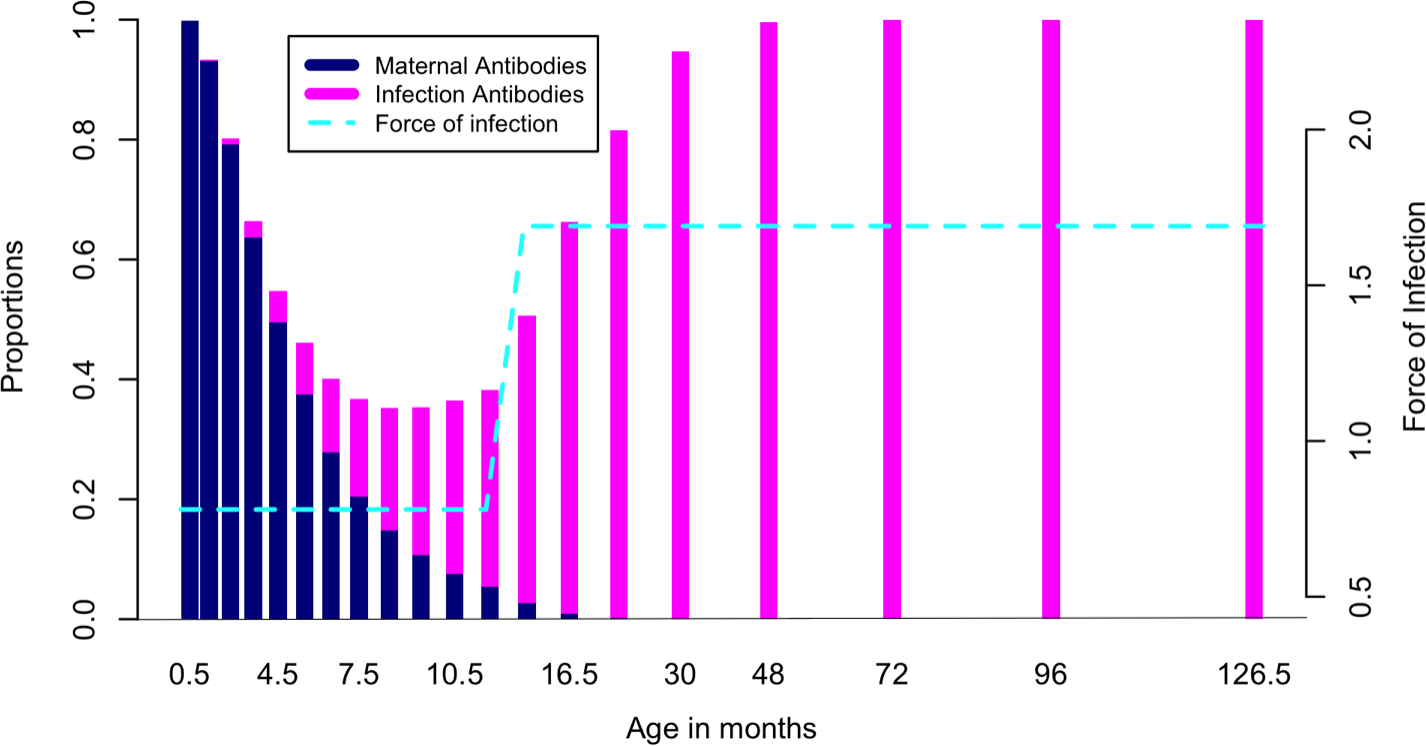
MSF Model predictions of the proportions seropositive and the force of infection acting on the different ages. The dark blue bars show the proportion at different age groups that have maternally acquired antibodies while the pink bars show the proportion that have been infected and hence have infection acquired antibodies, as predicted by the MSF model. The dashed blue line shows the stepwise force of infection function.

The MSF model gave a rate of loss of maternal antibodies of 2.54/year (95% CI 2.30–2.90) and a force of infection of 0.78/person/year (95% CI 0.65–0.97) for ages 0–1 year and 1.69/person/year (95% CI 1.27–2.04) for ages 1–12 years. (Fig 4) shows the fit for the MSF, and the 95% confidence region as established from refitting the model to the bootstrapped data. From these results, the average duration of maternally acquired antibodies (*D*_*M*_) is 4.72 (95% CI: 4.14–5.22) months, calculated as the reciprocal of the rate of decay. The average age at primary infection (*A)* is 15.1 (95% CI 12.7–18.5) months, while average optimal age to vaccinate is 6.8 months (95% CI 6.2–7.5). Model predictions of the proportions seropositive and the force of infection acting on the different ages are shown in (Fig 5).

The samples included in this analysis were from hospitalized children with different discharge diagnoses. Included in these were children diagnosed with LRTIs, a subset of which were RSV antigen positive. To check if the inclusion of these samples led to any bias, the MSF model with stepwise force of infection was refitted to 2 subsets of the data; excluding the LRTIs and the RSV antigen positives. The parameter estimates from these fits were not different from those obtained using all the data. This is shown in S2 Fig in S1 Text.

The model structure was modified to relax the assumption that the presence of maternal antibodies is refractive to infection. The force of infection acting on children prior to loss of maternal antibodies was estimated as 0.0/person/year, indicating that the data does not support this infection process (results not shown). The cut-off for seropositivity for ages 0–6 months was varied to try and account for differences between maternally acquired and infection acquired antibodies. We explored a range of cut-offs from 1.5 to 2, in steps of 0.25. Increasing the cut-off resulted in an increase in the rate of loss of maternal antibodies and a decrease in the force of infection. The estimate for the recommended optimal age of vaccination however, retained a relatively narrow interval of 4.7–7.1 months with different cut-offs, results in (S2 Table in S1 Text).

## Discussion

Vaccine intervention is likely to be key in controlling severe disease associated with RSV infection. Prior to vaccine introduction, epidemiological parameters such as the force of infection and average age at primary infection would be important baseline information. Analysis of age specific seroprevalence data can inform on the force of infection in a given population in the absence of vaccination. In this study we present the maternal antibody prevalence decay profile and subsequent age specific acquisition of antibodies to RSV among children in Kilifi and estimate the force of infection, average age at primary infection and the optimal age for RSV vaccine delivery.

The use of randomly selected inpatient plasma samples of children from 0–12 years, did not introduce bias to the estimation of seroprevalence in this study as might be speculated. Exclusion of all participants with LRTI or RSV positive antigen confirmed by IFAT/molecular method did not have a major change on the seroprevalence profiles. Interestingly, analysis of data on diagnosis at discharge showed that 31% of the admissions selected for this study were due to LRTI, with 16% of the admissions with LRTI caused by RSV. This clearly shows RSV as a major cause of lower respiratory tract disease in children in this setting, consistent with previous findings [10, 27].

The serological prevalence for RSV varied with age and showed 100% seropositivity in samples from children in the first month of life, i.e. <1 month old. This percentage decreased to 25% in the 6–7 month age group before rising again to 100% in 3 year olds and remained so upwards to 12 years. Though the data does not extend to include adults, this profile is qualitatively similar to what would be observed for immunizing infections such as measles[29]. An RSV serological study by Cox et al [14] in Brazil obtained a similar profile with a data set that extended to 31–40 year age group. The quick rise in the percentage seropositive observed in our data is indicative of a high rate of infection in the present population. High rates of infection, attributed to frequent reinfections, have also been observed in other studies [30–32], implying that the results from our analysis could be generalized to other settings.

Interpretation of age-prevalence data for RSV can be complicated by several factors. Waning of antibodies post primary infection could mean a reversion of serostatus. There could also be age dependence in the rate of infection. We explored a variety of catalytic models based on various assumptions on the properties of RSV specific antibodies. Including age-dependent force of infection provided a better fit to the data with or without assuming serostatus reversion following primary infection. An interpretation of this result is that while waning antibodies following primary infection have been observed, the reinfection rate is so rapid that the data are unable to distinguish a model with or without the process. If, however, vaccination were to reduce the rate of transmission of the virus, then the effect on the age-prevalence profile might be more pronounced and influence interpretation of such data.

The simplest model (MSF) gave estimates of the average duration of maternally acquired antibodies (D_M_) of 4.7 months and the average age at primary infection (A) as 15.1 months. These were not very different from D_M_ = 4.42 months and A = 14.2 months obtained from the model that allows for waning of antibodies post primary infection (model MSFSF_1_). In comparison, a seroepidemiological study by Cox et al [14] on RSV data from Brazil estimated the duration of maternal antibodies at 3.3 months. This estimate was obtained by fitting an exponential function to the data. Ochola et al [16] obtained estimates for the duration of maternal antibodies of 3.7 months(112 days) using data from a birth cohort and similar ELISA methods to the present study. Kinyanjui et al [33] estimated the duration of maternal antibody protection in the range of 2–4.5 months using data from hospitalizations and an age-structured deterministic model of RSV.

Amaku et al [20] fit a catalytic model with an age-dependent force of infection function to serological data from Brazil, the same data used by Cox et al [14]. The analysis estimated an average age at infection of 19.08 months (95% CI 16.68–21.48) and the force of infection function peaked at about 0.9/person/year in the 2-year age group. The vaccination window (defined as the period between the mean duration of maternal antibody and mean age at first infection) suggested by the Brazil results from Cox et al and Amaku et al [14, 20] 3.3–19.1 months, is wider than, though roughly similar to, that suggested by our results 4.7–15.1 months. As shown by an analysis of measles age-serological data, the window of vaccination can vary by location even within the same income level [34]. Mclean and Anderson [34] established a vaccination window for measles of 3–13 months for Senegal (data from 1957) and a window of 6–38 months for Thailand (data from 1967).

An estimate of 0.0/person/year for the force of infection acting on children who still have maternally acquired antibodies is in line with studies that show that the infant IgG response following primary infection is very low [15, 16, 32], and in fact, Ochola et al [16] showed that the pristine rate of decay of maternal antibodies was not different from the rate of decay including the infants who had had an RSV infection. Estimating our model parameters while excluding the LRTI positive sample and the RSV positive samples gave pristine maternal antibody decay rates of 2.56/year and 2.45/year, not very different from 2.54/year obtained with the entire data set. Varying the cut-off for seropositivity for the 0–6 months age group resulted in maternal antibody durations similar to other studies, but did not drastically change the recommended optimal vaccination age.

The optimal time to vaccinate children to prevent infection would be at an age after they have lost maternally acquired antibodies (which might interfere with the vaccine) and before their first infection. Proportions positive for RSV antibodies begin to rise soon after 6 months of age due to infection, as such; delaying vaccination could result in missing out on a significant amount of preventable infection/disease. Looking at the age-seroprevalence profile suggests vaccination should be carried out at around 6 months when seropositivity is at its lowest. However using a more formal approach, catalytic models results suggest a period between 5 and 15 months. Given that the force of infection estimated for ages 1–12 years is double that for ages 0–1 year, implying greater infection risk after 12 months, it would thus suggest vaccination should occur between 5 and 12 months. The vaccination window established by our analysis overlaps with the vaccination window of between 5 and 10 months obtained by Kinyanjui et al using an age-structured deterministic compartmental model of RSV and data from the same location as our study[33]. The benefit of delaying vaccination proposed by the Kinyanjui et al [33] model arose from delaying to allow for waning of maternal antibodies but still early enough to precede the high force of infection in the second year of life. In addition the effect of reduced transmission in the community arising from later infant vaccination (herd immunity) would provide indirect protection from infection in the children too young to be vaccinated. The model by Kinyanjui et al is drastically different from the model used in the current analysis, however it is promising to observe that the recommended vaccination window is robust to different modelling approaches and data types.

The catalytic model used in the current analysis is relatively simple. Variations could be made to the structure to account for varied antibody dynamics. However, we found that there was no added benefit of including antibody loss post primary infection, or allowing for infection while seropositive for maternally acquired antibodies. Antibody dynamics, such as waning, that have been observed to occur at an individual level, were not supported by the model and data used in this analysis. For a better understanding of this, an analysis similar to one done by Kucharski et al [35], where model outputs are individual level titers, might be more appropriate. The present analysis adds on to a growing body of evidence on RSV infection dynamics and the recommendation for control strategies. Infant vaccination should be given to children between 5 and 12 months old. Depending on the duration of protection afforded, vaccination might need to be optimized by time of year, relative to a RSV epidemic timing. An analysis similar to the present one done after a vaccination program has been in effect could aid in quantifying the effectiveness, it is expected that the force of infection and average age at primary infection would decrease.

## Supporting information

S1 Data Data used to generate results. (XLS)

S1 Text Additional information of the analysis. (DOCX)
