# Supplementary material for: Defining the vaccination window for respiratory syncytial virus (RSV) using age-seroprevalence data for children in Kilifi, Kenya
Source: PLoS One. 2017 May 22;12(5):e0177803. doi: 10.1371/journal.pone.0177803 (PMC5439681; doi:10.1371/journal.pone.0177803)
Supplement: S1 Text — (DOCX) [file pone.0177803.s002.docx]

**SUPPLEMENTARY MATERIAL**

**Defining the vaccination window for respiratory syncytial virus (RSV) using age-seroprevalence data for children in Kilifi, Kenya.**

Joyce U. Nyiro^1¶^, Ivy K. Kombe^1¶*^, Charles J. Sande^1, 5^, James Kipkoech^1^, Patience K. Kiyuka^1^, Clayton O. Onyango^2^, Patrick K. Munywoki^1^, Timothy M. Kinyanjui^4^, D. James Nokes^1, 3^

1. KEMRI-Wellcome Trust Research Programme, Centre for Geographic Medicine Research-Coast, Kilifi, Kenya.
2. Kenya Medical Research Institute/ Centre for Disease Control and Prevention, Kisumu, Kenya.
3. School of Life Sciences and WIDER, University of Warwick, Coventry, UK.
4. School of Mathematics, University of Manchester, Manchester, UK.
5. Department of Paediatrics, University of Oxford, UK

^*^ Corresponding author

E-mail: [ikombe@kemri-wellcome.org](mailto:ikombe@kemri-wellcome.org) (IKK)

^¶^ Equal contribution

1. Justification for stepwise function

In order to check if the force of infection was age dependent, we plotted the negative natural logarithm of the proportion susceptible *-ln(S(a))* against the mid-point of each age group. This showed that the data can be split into two clusters at the point where age=1 year, as shown in S1 Figure. With this, we set the force of infection as a stepwise function for age steps 0-1 year and 1-12 years.

**S1 Figure. A plot of the negative natural logarithm of the proportion susceptible (-ln(S(a))) against the mid-point of each age group.** Ages 0-0.5 years were not used to check force of infection function. The dashed lines are not fitted

2. Details of model selection process

We explored different assumptions in three nested catalytic models and compared the fits to data; the comprehensive results are shown in S1 Table. Fitting the model with the M-class split into 2 resulted in a better fit than using a single M-class for all three models. 2 M classes mean that the distribution for the duration of maternal antibodies is Erlang with a shape parameter equal to 2. As such, longer durations of maternal antibodies have higher probabilities than shorter durations. The fits were compared using the AICc value where differences >4 are considered significant. The subsequent results are from models with 2 M classes. Initially, fitting the models with a constant (i.e. age-independent) force of infection, the most complicated model, MSFSF_2_, gave the best fit with a –log likelihood value of 316.0 and AIC value of 637.9. The AIC difference between the MSFSF_2_ and MSFSF_1_ was 1.5 indicating that with the current analysis, we cannot see a significant improvement by MFSFS_2_ over MSFSF_1_. These results are shown in S1 Table. Continuing the analysis we fitted a step-wise age-specific force of infection with two steps for ages 0-1 year and 1-12 years. This was applied to the MSF and MSFSF_1_ models only, since MSFSF_2_ has implicit age-related force of infection (λ_0_ and λ_1_ are allowed to differ). The stepwise force of infection function was inferred from a plot of the negative natural logarithm of the proportion susceptible, *-ln(S(a))*, against the mid-point of each age group, see S1 Figure. Allowing for this flexibility in the force of infection function resulted in the two models fitting the data equally well, AIC_C_ difference of 1. As such, the most parsimonious model, MSF, was chosen as the best model.

**S1 Table: Comprehensive result of the nested model fitting.**

| **Model** | **Assumptions** | | **Parameters** | | | | | **-LL** | **AICc** |
| --- | --- | --- | --- | --- | --- | --- | --- | --- | --- |
|  |  |  | **Fixed** | | **Estimated** | | |  |  |
|  | **Force of Infection** | **Number of M classes** | **p*** | **δ*** | **σ** | **λ_0_** | **λ_1_** |  |  |
| **MSF** | Constant force of infection by age | 1 | 0 | NA | 2.64 | 0.95 | NA | 334.5 | 673.0 |
|  |  | 2 | 0 | NA | 2.75 | 1.04 | NA | 319.2 | 642.4 |
|  | Stepwise age-specific force of infection | 1 | 0 | NA | 2.08 | 0.51, 1.99 | NA | 322.5 | 651.0 |
|  |  | 2 | 0 | NA | 2.54 | 0.78, 1.69 | NA | 314.3 | 634.6 |
| **MSFSF_1_** | Constant force of infection by age | 1 | 1 | 4 | 2.86 | 2.07 | 2.07 | 330.9 | 665.8 |
|  |  | 2 | 1 | 4 | 2.95 | 2.20 | 2.20 | 317.7 | 639.4 |
|  | Stepwise age-specific force of infection | 1 | 1 | 4 | 2.25 | 1.26, 3.72 | 1.26, 3.72 | 323.3 | 652.6 |
|  |  | 2 | 1 | 4 | 2.71 | 1.80, 3.10 | 1.80, 3.10 | 314.8 | 635.6 |
| **MSFSF_2_** | Constant force of infection by age | 1 | 1 | 4 | 2.66 | 1.47 | 3.43 | 329.3 | 664.6 |
|  |  | 2 | 1 | 4 | 2.78 | 1.53 | 3.98 | 316.0 | 637.9 |

p= proportion that loses antibodies post primary infection; δ= rate of loss of antibodies post primary infection; σ=rate of loss of maternal antibodies; λ_0_=primary force of infection; λ_1_=secondary force of infection; -LL=the negative log likelihood value, the lower the value the better the model; AICc= the second order Akaike information criterion. * These parameters are fixed (not estimated). All rates are per person per year.

**S2 Figure. Results of fitting different subsets of the data.** All the data (960 samples), the data minus the samples that had positive RSV antigen results (898 samples), the data minus the samples that had positive LRTI results (614 samples). Parameter estimates from fitting these data sets are; σ = 2.54, λ_0-1_= 0.79, λ_1-12_= 1.69; σ = 2.45, λ_0-1_= 0.83, λ_1-12_= 1.70; σ = 2.56, λ_0-1_= 0.95, λ_1-12_= 1.58, respectively.

**S2 Table. Results of fitting the MSF model with age-specific force of infection using different cut-offs for maternal seropositivity.** σ=rate of loss of maternal antibodies; λ_0_= force of infection; D_M_ =duration of maternally acquired antibodies, A= age at primary infection, Av=recommended age to vaccinate
